# Supplementary material for: Impaired pulmonary ventilation beyond pneumonia in COVID-19: A preliminary observation
Source: PLoS One. 2022 Jan 25;17(1):e0263158. doi: 10.1371/journal.pone.0263158 (PMC8789183; doi:10.1371/journal.pone.0263158)
Supplement: Attachment — Submitted filename: Revision - Response to Reviewers.docx [file pone.0263158.s001.docx]

Journal Requirements:

When submitting your revision, we need you to address these additional requirements.

1. Please ensure that your manuscript meets PLOS ONE's style requirements, including those for file naming. The PLOS ONE style templates can be found at

<https://journals.plos.org/plosone/s/file?id=wjVg/PLOSOne_formatting_sample_main_body.pdf> and <https://journals.plos.org/plosone/s/file?id=ba62/PLOSOne_formatting_sample_title_authors_affiliations.pdf>

>> The revised manuscript meets PLOS ONE’s style requirements.

2. Please note that PLOS ONE has specific guidelines on code sharing for submissions in which author-generated code underpins the findings in the manuscript. In these cases, all author-generated code must be made available without restrictions upon publication of the work. Please review our guidelines at https://journals.plos.org/plosone/s/materials-and-software-sharing#loc-sharing-code and ensure that your code is shared in a way that follows best practice and facilitates reproducibility and reuse.

>> We are able to open-source the main code, which will allow researchers to see the structure of how it runs and links with the other parts of the code. (Ref. 21, Github link and software version). We have also put the functions and libraries for model boundaries, IOs and data analysis in a public repository. This should allow researchers to start to replicate the work. Because there is commercial interest and involvement of more than one large organization in the development of the code, the intellectual property ownership is hard to disambiguate, and without extensive permissions the only thing we can’t open-source immediately are some of the functions. To get around this we have already started a project to work on an open-source version of this software, and plan to release in about 1-2 years.

3. Thank you for stating the following financial disclosure:

“The funders had no role in study design, data collection and analysis, decision to publish, or preparation of the manuscript.”

At this time, please address the following queries:

a) Please clarify the sources of funding (financial or material support) for your study. List the grants or organizations that supported your study, including funding received from your institution.

b) State what role the funders took in the study. If the funders had no role in your study, please state: “The funders had no role in study design, data collection and analysis, decision to publish, or preparation of the manuscript.”

c) If any authors received a salary from any of your funders, please state which authors and which funders.

d) If you did not receive any funding for this study, please state: “The authors received no specific funding for this work.”

Please include your amended statements within your cover letter; we will change the online submission form on your behalf.

>> We have stated followings for the financial disclosure.

------------

This work was supported by the Korea Medical Device Development Fund grant funded by the Korea government (the Ministry of Science and ICT, the Ministry of Trade Industry and Energy, the Ministry of Health & Welfare, Republic of Korea, the Ministry of Food and Drug Safety) (Project Number: 202011A03). The funders had no role in study design, data collection and analysis, decision to publish, or preparation of the manuscript.

4. Thank you for stating the following in the Competing Interests section:

“I have read the journal's policy and the authors of this manuscript have the following competing interests:

Soon Ho Yoon works as a chief medical officer in MEDICALIP Co. Ltd. All other authors do not have a conflict of interest to declare associated with this publication”

Please confirm that this does not alter your adherence to all PLOS ONE policies on sharing data and materials, by including the following statement: "This does not alter our adherence to PLOS ONE policies on sharing data and materials.” (as detailed online in our guide for authors http://journals.plos.org/plosone/s/competing-interests).  If there are restrictions on sharing of data and/or materials, please state these. Please note that we cannot proceed with consideration of your article until this information has been declared.

Please include your updated Competing Interests statement in your cover letter; we will change the online submission form on your behalf.

>> We have included following Competing Interests statement in the cover letter.

------------

I have read the journal's policy and the authors of this manuscript have the following competing interests: Soon Ho Yoon works as a chief medical officer in MEDICALIP Co. Ltd. All other authors do not have a conflict of interest to declare associated with this publication. This does not alter our adherence to PLOS ONE policies on sharing data and materials.

5.  In your Data Availability statement, you have not specified where the minimal data set underlying the results described in your manuscript can be found. PLOS defines a study's minimal data set as the underlying data used to reach the conclusions drawn in the manuscript and any additional data required to replicate the reported study findings in their entirety. All PLOS journals require that the minimal data set be made fully available. For more information about our data policy, please see http://journals.plos.org/plosone/s/data-availability.

Upon re-submitting your revised manuscript, please upload your study’s minimal underlying data set as either Supporting Information files or to a stable, public repository and include the relevant URLs, DOIs, or accession numbers within your revised cover letter. For a list of acceptable repositories, please see http://journals.plos.org/plosone/s/data-availability#loc-recommended-repositories. Any potentially identifying patient information must be fully anonymized.

Important: If there are ethical or legal restrictions to sharing your data publicly, please explain these restrictions in detail. Please see our guidelines for more information on what we consider unacceptable restrictions to publicly sharing data: http://journals.plos.org/plosone/s/data-availability#loc-unacceptable-data-access-restrictions. Note that it is not acceptable for the authors to be the sole named individuals responsible for ensuring data access.

We will update your Data Availability statement to reflect the information you provide in your cover letter.

>> All relevant data are within the manuscript. The institutional IRB allowed the analysis of CT images for this study solely, so CT images used in this study cannot be shared based on the IRB approval.

6. We note that you have stated that you will provide repository information for your data at acceptance. Should your manuscript be accepted for publication, we will hold it until you provide the relevant accession numbers or DOIs necessary to access your data. If you wish to make changes to your Data Availability statement, please describe these changes in your cover letter and we will update your Data Availability statement to reflect the information you provide.

>> All relevant data are within the manuscript. The institutional IRB allowed the analysis of CT images for this study solely, so CT images used in this study cannot be shared based on the IRB approval.

[Note: HTML markup is below. Please do not edit.]

Reviewers' comments:

Reviewer's Responses to Questions

**Comments to the Author**

**Reviewer #1:** The authors presented a retrospective study of analyzing the relationship of ventilation defect with pneumonia by CT-based FAN flow model for COVID-19 patients. Their results demonstrated the availability to assess functional impairment in COVID-19 from the integration of clinical imaging with computational modelling. In addition, the diagnostic accuracy in identifying dyspneic patients by the ventilation defect analysis with the FAN was also satisfying. The study addresses an important issue and the results are inspiring for clinicians. Some questions still remain to be cleared.

1, Since the FAN flow model is the foundational methodology of this work, the authors should provide more details about the flow calculation (may be in the supplementary material), such as the flow equations, model hypothesis, simulation method and so on. I understand that the modeling details have been described in their published studies as they referred to. However, it is better to explain the basic methodology in this paper for the readers who know the FAN model for the first time.

>> We have added governing equations of the FAN model in the manuscript.

------------

(Line 194-203)

Under an assumption of the insignificant inertial force during the normal breathing cycle, the flow in a single airway compartment can be calculated as

$Q_{d}=\frac{P-P_{d}}{R}+\frac{C}{2}\left( \frac{dP}{dt}+\frac{dP_{d}}{dt} \right)$,	(1)

were $Q_{d}$ is the flow rate in an airway, $P$ and $P_{d}$ are the nodal pressures, and $R$ and $C$ are the airway resistance and compliance, respectively. If we assume acinar deformation over time $t$ is isotropic, the equation of acinar dynamics is formulated as

$I\frac{d^{2}V_{a}}{dt^{2}}+R_{a}\frac{dV_{a}}{dt}+\frac{V_{a}}{C_{a}}=P_{a}-P_{pl}$,	(2)

where $I$ is the inertance of acinar motion, $V_{a}$ is the volume of an acinus, $R_{a}$ is the resistance of acinar deformation, $C_{a}$ is the acinar compliance, $P_{a}$ and $P_{pl}$are the intra-acinar pressure and the pleural pressure, respectively.

2, Is there a better way to display the results in Fig. 4? For example, the panels can be arranged based on the different groups.

>> Thanks to the reviewer for this comment. We have updated Fig 4 with the newly arranged panels based on the different groups.

3, The authors declared that there was an outlier (x=1.2%, y=7.8%) in Fig. 5. However, there are two more outliers in the figure, one is around (18%, 3%), the other is around (19%, 29%). Is there any analysis about this result?

>> The current analysis includes all data points including outliers (without the outliers, y=0.89x + 2.06 and R^2^=0.94). The outliers demonstrate the existence of disagreement between structural abnormality (ex: % pneumonia) and functional impairment (ex: %ventilation) as shown in previous lung studies (Cerveri et al., Chest 2004; Rabe et al., Am J Respir Crit Care Med 2017; Yahaba et al., Eur J Radiol 2014). In this figure, we call attention to one outlier which shows large ventilation defects (functional impairment) even though minor pneumonia (structural abnormality). But as shown in the discussion, we have left its confirmation for follow-up studies with a larger cohort.

4, In Fig. 6a & b, the authors showed the percentage ventilation and ventilation CV with different lobes indicated by different colors. The correlations showed in the text, however, were given by the total data. How are the correlations between ventilation with pneumonia on different lobes?

>> In this pilot study with a small number of samples, we used lobar parameters and present the correlation using all data points instead of comparing the lobar correlations. Similar lobar analyses have been presented in multiple previous lung image studies (Matin et al, Radiology 2016; Doganay et al., Eur Radiol 2019).

5, Some typos. For example, Page 16, Line 355, 356, extra space.

>> Thanks to the reviewer to find this. We have corrected those typos.

**Reviewer #2:** The authors reported interesting findings from a pilot study performed on patients with COVID-19 (n=25) and healthy controls (n=4). They provided a post-processing analysis to quantify regional lung ventilation. Dyspnoic CVOID-19 patients had both higher anatomical involvement and larger ventilation defect as compared to non-dyspnoic patients. A direct correlation linked anatomical involvment with ventilation defect; a single patient had a moderate non-concordance (eg higher ventilation defect as expected by the anatomical involvmente). This is a well performed stimulating study, very appealing from a technical point of view. My main concern regards the authors' hypothesis, that though intriguing, should be partly downgraded in light of the study result; indeed, the evident finding from the present study is the robustness of the FAN technique in the evaluation of COVID-19 pneumonia as compared to the traditional anatomical evalutaion. However, the possibility of FAN to provide additional information, though plausible, relys on a single outlier and should be more carefully reported.

>> We agree with the reviewer's comment. We have tried not to draw to strong conclusion from this small pilot study, and as a consequence of the reviewer’s suggestion, the followings have been added to the discussion.

------------

(Line 368-372)

We have demonstrated a case with a relatively large ventilation defect area compared to the degree of pneumonia identified on CT. This might indicate a cause of breathlessness in patients with less severe pneumonia on CT. However, the number of cases presented in this pilot study is small. Additional studies involving larger cohorts are necessary to confirm this.

>> Additionally, conclusion section has now been reworded to address reviewer’s comments.

------------

(Line 383-391)

In conclusion, this study has used a CT image-based FAN model to investigate impaired ventilation induced by pneumonia in COVID-19 lungs. The FAN model was potentially capable of deriving regional ventilation and dynamic airflow impairments in dyspnea and symptomatic patient groups. Although the FAN model has focused on the computational evaluation of ventilation defects, its sensitivity and specificity were comparable to the extent of pneumonia identified on CT. In addition to using ventilation abnormalities identified with the FAN model, including perfusion from CTPA scans and the consequent ability to model gas-exchange could potentially help with understanding the pathophysiology and profound hypoxia that symptomatic patients experience.

Minor comments:
- If available, some few more data could be reported (i.e. dimer level, CRP, P/F etc. and their correlation with CT findings)

>> We have added CRP Whole blood cells, Lymphocyte, Lactate dehydrogenase, D-dimer and Partial pressure of arterial O2 in the table 1.

- Figure 5 could be displayed with dots of different colour according to the underlying study group (asymtpomatic, dyspnoic etc)

>> Figure 5 has been updated with different colour according to the study group.
